# Measuring User-Perceived Characteristics for Banking Services: Proposing a Methodology

**DOI:** 10.3390/ijerph19042358

**Published:** 2022-02-18

**Authors:** Olga Vl. Bitkina, Jaehyun Park, Hyun K. Kim

**Affiliations:** 1 Department of Industrial and Management Engineering, Incheon National University (INU), Academy-ro 119, Incheon 22012, Korea; olgabitkina@inu.ac.kr; 2 School of Information Convergence, Kwangwoon University, Kwangwoon-ro 20, Seoul 01897, Korea

**Keywords:** banking services, user experience, perceived trust, perceived usefulness, perceived ease of use, perceived security, perceived convenience

## Abstract

With the continuous technological enhancement of banking services, customers can avail of better, more secure services which present improved opportunities and convenience. Of the many methods available to perform banking operations, customers commonly use traditional banking, online banking, and mobile banking. Each of these existing methods has advantages and limitations that affect customer experience, trust, satisfaction, and continued intention to use such services. In this study, an attempt was made to develop and fit a model to evaluate and measure the effect of perceived characteristics on banking services. To this end, a questionnaire was administered to 91 participants in Korea to investigate their experiences in the three types of services: offline banking (traditional banking), online banking, and automated teller machines (ATM). The factor design for evaluating the user experience through the perceived characteristics of the banking system was performed by conducting exploratory and confirmatory factor analyses. The proposed model exhibited validity and reliability to evaluate the user experience in the banking system. The results obtained can help banking specialists and professionals increase the level of customers’ trust, loyalty, and intention to use their services.

## 1. Introduction

As a component of the financial sector, banking is constantly undergoing technological changes [1]. Service and product use depend on customer- (user) perceived characteristics and their combinations in different industries, including banking [2,3,4,5,6,7,8,9,10]. Moreover, the loyalty of the consumer to a service or product makes him return to the same provider again, which is the key to business success in the modern world of competition [10]. Previous studies [2,3,4,5,6] showed that a loyal consumer is willing to pay the same provider, again strengthening its position in the market. This is especially important in the financial sector, where many financial transactions are hidden from the user (for example, an online service). It should also be noted that the user’s loyalty supports his readiness to adopt new technologies, which ensures the overall technical progress of society. Based on this, this research purpose is of particular importance for the producers of goods and services in order to ensure a sustainable income and also for the technical development of society as a whole, ensuring the introduction of new financial and production technologies [4,9]. Global tendencies in the frequency of usage of traditional and modern bank services were reported [11]. The results were obtained through a survey, encompassing 35,642 consumers from 43 different countries. The most commonly used services are online/offline banking and ATMs—preferred by more than 85% of users. Mobile banking and call center services are less popular, with 35–40% of consumers never having used them. Progress in banking services is closely related to technology development; new technological solutions provide bank customers with convenient and fast services such as online banking, mobile banking, and ATMs. However, each service also has its own peculiar disadvantages, which affect customer trust, loyalty, and the customer’s intention to use a bank service provider or the service itself [12,13]. The main advantages and disadvantages of these banking services have been examined by some previous studies [14,15]. Online banking is an easily accessible service with high mobility, easy setup/use, and fast transactions. Simultaneously, this service has transaction-type limitations and security/privacy issues, fosters opportunities to make rash and hasty expenses, and lacks the facility of assistance. Offline banking provides users with various transaction-type options and prompt personal assistance but costs time and effort, with the inevitable requirement for documentation or paperwork. ATM services are characterized by relatively easy access, with fast transactions and some disadvantages such as a service charge, the lack of assistance, the time and effort spent finding an ATM, security/privacy issues, and transaction-type limitations. These aspects affect the quality of the user experience and thus the intention of service use.

User experience in different products and services, including the banking sector, is an important element affecting the perception and intention of use [16,17]. Results from studying user experience in Internet banking supported the positive relation between perceived trust (PT) and accepting or using Internet banking services [18]. The banking system was studied, with attention to mobile banking [19,20], and it was found that system and information quality are connected to customers’ trust and satisfaction. These findings show that changes in users’ trust lead to changes in satisfaction and banking service perception. Based on this, the main research question of this paper is to understand the mechanisms for evaluating the user experience of banking services from the consumer’s perspective. This indicates that studying user experience and the interaction between users and service providers, including perceived characteristics, is an important issue that helps develop and improve banking services. Research in this area entails benefits for stakeholders (banks and customers) and can contribute to the development of the banking system.

The present study aims to understand the relationship between perceived characteristics in banking services and to extract a mechanism of user experience evaluation in online, offline, and ATM services. Perceived trust, security, convenience, ease of use and usefulness (PT, PS, PC, PEU, and PU) are considered to be factors affecting customer experience. The most dependent perceived characteristics on the type of banking service are also shown. The model thus developed proposes new knowledge and findings to help banking specialists and researchers improve service attractiveness among customers and increase trust in new banking technologies.

The results expected in the course of this study may contribute to the problems of increasing the competitiveness in the financial sector among service providers, expanding the choice of convenient and reliable services for the consumer, as well as ensuring faster and more reliable implementation of new technologies in banking. In turn, the introduction of new technologies in the financial sector will contribute to the overall technical development of society.

## 2. Perceived Characteristics of Banking Services

The present study evaluates user behavior in three predominant, different types of banking services (online, offline, and ATM) through PT, PS, PC, PEU, and PU. Based on previous studies, PT includes users’ expectations of system performance [21,22]. PS is the customers’ perception of the degree of protection against these threats [23]. PC is the extent to which an individual believes a product (service) will enhance simplicity [24]. PEU is the degree to which a person believes that using a particular system would be free of effort [25]. PU is the degree to which a person believes that using a particular system would enhance his/her job performance [25].

For the presented study, measurement methods and tools of the user-perceived characteristics were selected from previous studies and guidelines that have proven their validity [2,3,4,5,6,7,8,9,10]. The mentioned research showed that the service and product introduction (adoption) depends on customer- (user) perceived characteristics and their combinations in different industries, including banking, trade, stocks, cryptocurrency, payments, and online wallets. The standard methods for collecting data and evaluating them among users are surveys, interviews, experiments involving human objects with simulations of various scenarios. The analysis of the obtained data is carried out by different standard approaches: ANOVA, t-test, factor analysis, descriptive analysis, regression, and mathematical modeling. The number of participants in surveys, interviews, and other experiments varies but usually exceeds perceived 90. The above statistics on the tools for measuring the characteristics perceived by users are summarized in Table 1.

Based on Table 1, an analysis approach was built to obtain valid results using a survey with a Likert rating scale. The Likert scale is a valid and commonly used assessment tool in surveys [26,27]. The main benefits of choosing the Likert scale are a wide response rate, increased analytical results, the possibility of a neutral opinion, and the reasonable time for a survey and data analysis. Based on the statistical data, the above assessment and measurement tools were selected to perform factor analysis.

### 2.1. Perceived Trust

PT in different types of banking services is a core element of the user experience and can be represented through various concepts and constructs (Table 2).

Two concepts of trust-affecting factors (vendor- and medium-based) were proposed [33]. Vendor-based factors are related to the trustee party, and medium-based factors to the medium of communication between parties. This hypothesis was confirmed by the finding that states that trust is an influential factor in strengthening customers’ intention to use banking innovations [20]. Previous studies [34] found a positive role of PT in banking services. A positive connection between trust and technology has also been discussed [35,36]. Scholars who examined Internet banking found that trust is an important factor for the successful implementation of new technologies in the service [37]. PT in banking service providers is directly connected with the level of trust generated by the service.

Different perceived characteristics can relate to and represent PT in banking. It was confirmed that consumers’ trust in online technologies has a positive relationship with PEU and PU [38]. A positive influence of PS and PT on the frequency of product use was found [39]. Relationships between trust in the product (service) and trust of the manufacturer (provider) in mobile and online technologies were found [40,41,42,43]. PT has a direct connection with actual trust and can be mathematically described by the relationship between the users’ (consumers) trust, credibility, reliability, intimacy, satisfaction, and the self-orientation of the manufacturer [44]. Considering all these reasons, the present study proposes PT as the predominant factor for evaluating user experience in different types of banking.

### 2.2. Perceived Security and Convenience

A core research issue in electronic services (including online and Internet banking) is the PS level, because the use of a partially invisible service gives a feeling of uncertainty to users, with possibilities rife with system failures or unauthorized access. PS is dependent on online transmission conditions in electronic services [23]. Scholars have described PS as a user’s belief in the protection of his/her transactions from any threats; moreover, PS affects customer willingness to interact with bank accounts [45]. PS is one of the most important factors in online banking services and is associated with the user interface [46]. This is explained by the consumer’s desire to render the process of using these services more transparent and comprehensible through the appropriate interface. PS increases with PT. Trust in banking is “the subjective probability with which customers believe that a particular transaction occurs in a manner consistent with their confident expectations” [47]. Therefore, building trust is helpful to reduce security threats and in adapting to banking technologies [48].

Time and effort are the basic characteristics of service convenience [49]. Convenient services can be characterized by the ability to save customer time and minimize the expending of physical and emotional efforts to perform tasks [49]. Taking PC into account, user behavior in various services was examined [50]. As a result, PS and PC were included in the basic model confirming their connection with user experience.

### 2.3. Perceived Ease of Use and Perceived Usefulness

PEU and PU are related to the productivity and efficiency of the service and can be studied simultaneously in relation to user experience [51]. Task performance and PEU are linked through the ability of a product or service to increase the speed, productivity, and efficiency of user activity [52]. A positive relationship between PEU and the long-term intention to use banking services was confirmed by taking PU into account. The technology acceptance model (TAM) and its elements in banking services were studied [53]. PEU and PU are shown as integral parts of TAM that influence user behavior intentions toward banking services. Numerous studies show that PEU and PU are important factors influencing user experience, not only in the banking sector but also in many others (Table 3).

## 3. Materials and Methods

### 3.1. Participants

For the experiment, we randomly selected 91 users of the banking system in Korea (47 males, 44 females). The average age of the participants was 30 (standard deviation: ±5 years). All the participants were regular users of online/offline banking and ATM services with over 5 years’ experience. The purpose and procedure of the survey were explained to the participants before the experiment. Participation in the experiment was voluntary.

### 3.2. Experiment

#### 3.2.1. Questionnaire

In the experiment, user attitude and experience in service were analyzed through PT, PU, PEU, PS, and PC according to the types of banking services (offline, online, and ATM). The questionnaire (Appendix A) was developed based on previous research on the perceived characteristics in services [58,59,60,61,62].

#### 3.2.2. Experimental Procedure

The questionnaire was administered to the 91 participants, and each of them completed the survey within 1–2 days. The questions were not shown to the participants in advance. The evaluation of each banking service (online, ATM, and offline) by the users was performed using a scale from 0 (lowest) to 10 (highest). During the initial assessment of the results, the questionnaire submitted by one participant was removed from subsequent analysis due to invalidity; the survey was completed in a matter of a few minutes, and each answer was rated 10 points. It was obvious that the participant did not respond carefully.

### 3.3. Analysis

In the present study, exploratory and confirmatory factor analysis (EFA and CFA) were used simultaneously. Results from the EFA were considered as pre-established theory, and the CFA was applied to confirm the extracted factors and the item structure from EFA [63]. This analysis approach has proven its validity in previous studies [64,65]. EFA is effectively used to identify and exclude empirically redundant members of the questionnaire. This method can also be applied to study and adjust the measure dimensions during the survey. In turn, DFA is used to confirm the developed model and prove the research hypothesis. Previous studies [64,65,66] showed that factor analysis can be one of the most effective assessment methods in the behavioral and social sciences.

EFA was performed using SPSS software (IBM Corp., New York, USA) to identify the relationships between the measured variables and to determine the underlying structure of the existing set of variables. The initial factor model contained sets of manifest variables (PT1-PT9; PS1-PS9 etc.), which are functions of the common factors (PS, PC, PT, PU, PEU). The Kaiser–Meyer–Olkin measure and Bartlett’s test were used to evaluate the suitability of the data set for the factor analyses. EFA was performed using Varimax rotation, which allowed the extraction of the optimal three-factor solution. Common factors influenced the set of manifest variables, and factor loadings were obtained to measure the impact of the common factors on manifest variables.

CFA was performed using AMOS software (IBM Corp., New York, USA) to verify the factor structure based on manifest variables. The CFA used in this study is based on a three-factor solution proposed by EFA, and the results showed existing relationships between the factor and observed variables. Satisfactory results with goodness-of-fit (GOF) indexes were obtained.

## 4. Results

### 4.1. Exploratory Factor Analysis

The Kaiser–Meyer–Olkin Measure of Sampling Adequacy showed satisfactory results with a value of 0.824. The Bartlett’s Test of Sphericity gave a *p*-value <0.000. The Kaiser rule [67] was applied to select factors with eigenvalues starting from 1.0. The results of EFA suggested a three-factor model based on Varimax rotation of the studied perceived characteristics displaying the highest correlations (Table 4).

Table 3 shows selected items with the highest correlation (loading) with factors. Item (variables) selection was performed using the below criteria [63]:

− Selecting questionnaire items whose loadings were above 0.5;
− Screening out factors whose questionnaire items were less than 2;
− Screening out questionnaire items whose loadings were above 0.5 in a few factors at the same time. Based on the above criteria, the three-factor model was extracted. The PT factor had four items; the PS and PC factors had three items. Cronbach’s alpha showed values above 0.65 for each factor, which indicated satisfactory correlations between a set of items as a group [68,69].

### 4.2. Confirmatory Factor Analysis

**Figure 1:**
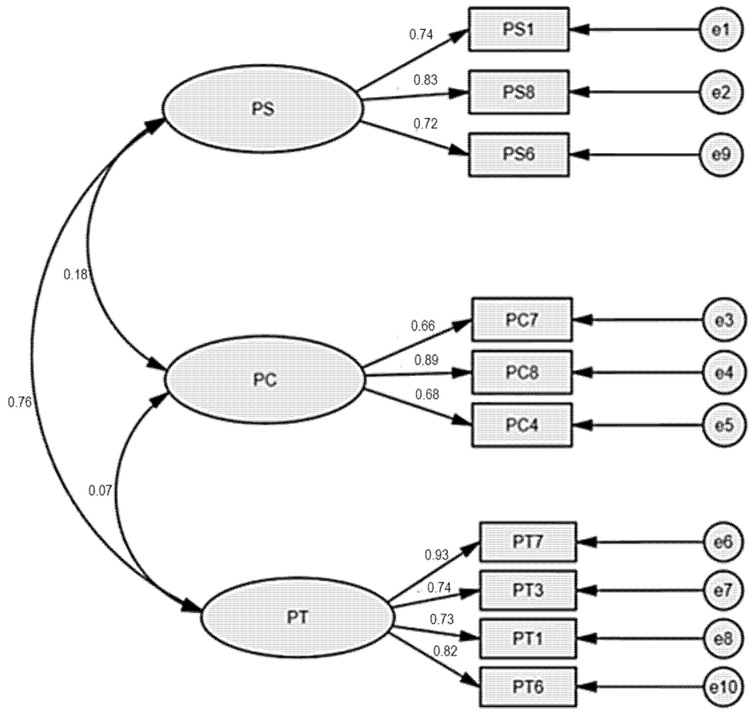
Factor Loading CFA Results.

The extracted three-factor model (Table 4) was analyzed through CFA, and the results are show in Figure 1, with the main goodness-of-fit indexes shown in Table 5.

Figure 1 shows that factor loadings were above 0.5 and indicates that there were satisfactory correlations between factors and extracted variables [70,71]. Model goodness-of-fit indexes (Table 5) showed satisfactory results, GFI and CFI >0.9; NFI >0.85; CMIN/DF <2 and RMSEA <0.08. Generally, EFA and CFA showed acceptable results and demonstrated the adequacy of the developed three-factor model assessment of perceived characteristics in banking services (Table 6).

Three-factor models have already shown their effectiveness in previous studies in different areas such as trade, shopping, medicine. A study [66] applied the CFA to psychological assessments for Beck Depression Inventory scores. An effective three-factor model was proposed including negative attitude, performance difficulty, and somatic factors. Customer service perception in multiple areas through a three-factor structure was studied [72]. The authors proposed customer service orientation, flexibility, and assertiveness as the main factors. Previously developed three-factor models have proved their efficacy in different research fields.

## 5. Discussion

The developed model unites PS, PC, and PT factors which allow the measurement of user attitude and experience. To obtain a valid model, a measurement instrument of perceived characteristics (questionnaire with scale) was compiled based on the recommendations of various educational institutions and government organizations. The main guidelines for constructing questionnaires were discussed in previous studies [73,74]. The following general principles were applied:

− Selection of a proven previous study on a similar topic as a prototype (Appendix A);
− Questions should be clear and direct;
− Only one question can be asked at a time;
− It is necessary to avoid bias and verbosity in matters;
− The measurement scale should provide a differentiated assessment of the expressed opinion.

Existing research provides evidence that these factors are mutually influential. It was shown [75,76] that in a user experience evaluation, PT in a service or a system relates to PS and PC. Studies considered the user-centered approach and found that the establishment of trust in society and in a service is significantly important and affects the user experience together with their attitude toward the service. In turn, PT relates to PS by linear dependency between the users’ sense of trust and security in their transactions and the financial activity involved in the service [45]. It is important to provide the PT and PC connection for the growth of the service (product) attractiveness among potential users [29]. This is because customers consider PC in terms of the time and effort spent, which are important factors influencing user experience together with trust [77]. Moreover, customers are convinced that using any of the three banking services (online, offline, or ATM) takes time and effort, albeit in different proportions. Thus, these studies do not differentiate much between the degree of convenience for different types of banking but evaluate this in general for the banking system. PS and user experience are connected through the intensity of service usage [78,79]. The present study considers regular users of the studied banking types/services and observes that because of their extensive experience of use, consumers believe that these types of banking are safe and thus do not see differences in security among them. This explanation is based on studies confirming that extensive use experience increases the PS of the service in general. Thus, our model finds confirmation of its effectiveness in previous studies that show a mutual connection between user experience, PT, PC, and PS in various types of banking.

PEU and PU did not show a significant connection with user attitude and experience in banking services. Previous studies have shown similar results in various research and service areas. A study [54] found that PEU and PU do not significantly affect the intention to use mobile banking. A healthcare research [80] showed that PEU is not significantly correlated with attitudes toward the use of personal health record technology. A study [81] showed an insignificant effect of PEU on the intention to use different technologies. The insignificant influence of PU on actual technology use was demonstrated [82]. Based on previous studies [83,84], these results can be related to the fact that users are cautious with all banking operations related to financial transactions. In this case, PEU and PU are of secondary importance compared with other factors (especially PS and PT), when it comes to the assessment of user attitude, intention, and experience. The present study considers all frequently used banking services, including online service. Despite the familiarity with online technologies, their use in financial transactions is still perceived as unpredictable and unreliable [83]. This statement describes only PEU and PU and is contradictory to the results for the primarily important factor of PT, because PT is based on the perception of reliability and confidence in the service used. Based on this, the final model included the most important factors, but PEU with PU were not accepted by the respondents as primary ones.

One of the important issues related to banking services is the verification of the degree to which existing services meet the user’s requirements and expectation [19,20]. The development of such evaluation models that include all types of popular services is important for new banking service adoption and perception among users [30,32]. Studies show that items related to the different types of banking influence the perception of service, type of experience in the service environment, and intention to use the product or service [16,17]. Moreover, technology and product acceptance depend on PT, PS, and PC [45,49], which confirms this study’s general results and findings. These studies illustrate that the type of banking service and its perceived characteristics are factors influencing new service and technology introduction. In contrast to previous studies, the present research helps unite the items related to the different types of banking in one model.

The performance of the developed model was supported by satisfactory goodness-of fit indexes including the ratio CMIN/DF, the comparative fit index (CFI), the goodness-of-fit index (GFI), and the mean-square error of approximation (RMSEA) proposed by [85]. A study [63] developed a questionnaire for evaluating the user value of mobile devices through EFA and CFA. It reported satisfactory CFA indexes, i.e., GFI = 0.910, CFI = 0.9, NFI = 0.85, CMIN/DF = 2.664, and RMSEA = 0.075. A CFA of a five-factor model [86] for a questionnaire of online training evaluation in the workplace was performed and provided the index values of GFI = 0.936 and CFI = 0.873, as well as RMSEA = 0.067. According to previous studies, the proposed model has a satisfactory performance according to GOF criteria.

Generally, the presented model shows effectiveness and reliability; however, there are a few limitations to be improved in future. Firstly, using qualitative data with quantitative data would increase the versatility of the model. The evaluation of subjective measurements such as PT, PC, and PS can be improved by prospect theory application based on a previous study [87]. This theory can be successfully applied to compensate for personal decision-making behavior in uncertain situations.

## 6. Application, Limitations, and Future Research

The three-factor model with extracted units of PS, PC, and PT factors was obtained and allowed the measurement of user attitude and experience in financial and banking industries. Based on the above research, the results may contribute to the problems of increasing the competitiveness in the financial sector among service providers, expanding the choice of convenient and reliable services for the consumer, as well as ensuring faster and more reliable implementation of new technologies in banking. In turn, the introduction of new technologies in the financial sector will contribute to the overall technical development of society.

The presented study has several limitations, which can be improved in future research. Limitations of this study include the number of banking services analyzed (offline, online, and ATM). In the future, the types of services will be expanded for analysis, including mobile banking and different banking electronic apps for customers. The developed model refers to the current period of using the banking service. The model can be extended to include past user experience with the banking service. The methodology can be expanded by including data from qualitative interviews with respondents. Analysis approaches can be supplemented by statistical data about survey participants (for example, gender, age, occupation) in the model variables, using regression and statistical methods. Participants can be divided into groups according to the type of professional activity (specialist in the financial sector or not), work experience, as well as experience in using banking services, for a more detailed analysis.

## 7. Conclusions

The present study proposes a methodology that can be helpful to evaluate user experience, attitude, and intention through PT, PC, and PS in different banking service types. The EFA allowed the extraction of 3 factors and 10 items (variables) for the developed model. CFA supported the validity of the model through satisfactory factor loading and GOF values. It was shown (Table 6) that protection from threats in online banking, confidentiality in offline service, and ATM security are important for PS growth. In turn, time and knowledge spent by a user are the main factors for PC improvement. PT can be increased if the service provider will support service reliability, clarity, and fulfillment of commitments. These principles can be used by financial professionals and banking experts to strengthen their customer base and develop an improved service for their business.

These findings are important for new banking technology implementation supporting progress and development in the banking sector. The factors detected are motivators for the customer response toward new banking technologies and different service types. The obtained results can be extended to different types of services, such as online shopping and trade. The presented highlights can be used as guidelines to determine customer behavior, attitudes, and their perceptions toward services. The findings can offer researchers, service providers, and sellers assistance in understanding how to build customer trust and security in new types of services and ensure long-term loyalty. As it was demonstrated above, improving the quality of services is associated with strengthening customer satisfaction and expanding the customer base. In turn, customer loyalty and satisfaction help to introduce new types of services using new financial and technical innovations. Thus, the conducted research can be used locally in the banking and financial sector and help in the development of new technologies for the society as a whole.

## Figures and Tables

**Table 1:** Measuring tools of the user-perceived characteristics.

Perceived Characteristic	Service/ Product	Methods/Approaches	Sample Size	References
Perceived Trust	Online and Offline Banking, Trade, and Stocks	(1) Survey and Interview with ANOVA, *t*-test, and factor analysis (2) Other experiments involving human subjects with ANOVA, *t*-test, factor/descriptive analysis, regression, and mathematical modelling	About or over 100 participants	[2,3,4]
Perceived Security	Online and Offline Banking, Trade, and Stocks Mobile Payment and Wallets			[2,3,4,6]
Perceived Convenience	Online Banking, Trade, Cryptocurrency			[4,5,6,9]
Perceived Ease of Use	Online Financial Services			[8,10]
Perceived Usefulness	Online and Offline Financial Services			[2,3,6,8]

**Table 2:** Relations of PT and other constructs.

References	Type of Banking Service	Constructs	Methods	Brief Findings
[28]	Internet banking	Provided information, e-banking system, the website of a bank, a bank’s characteristics	Logistic regression analysis	The most powerful factor in the trust-building process is the e-banking system and the website
[29]	Online banking	Perceived security, usability, reputation, commitment of clients	Regression analysis	Security, privacy, usability, commitment of clients and reputation have significant association with PT
[30]	Internet banking	PU, PEU, perceived financial risk, perceived security risk, attitude to using, behavioral intention	Structural equation modeling	Security and financial risks are negatively related to PT
[31]	E-commerce	E-commerce knowledge, perceived reputation, perceived risk, perceived technology	Partial least squares–Structural equation modeling	e-commerce knowledge, perceived risk and perceived technology have significant influence on PT
[32]	E-commerce	Word of mouth, online experience, security/privacy, perceived risk, brand reputation, quality information	Multiple regression analysis	Security/privacy, word of mouth, online experience, quality information, and brand reputation have a significant and positive relationship with PT

**Table 3:** PEU and PU as items representing constructs.

References	Type of Service	Represented Construct	Methods	Brief Findings
[54]	Mobile banking	Intention to use mobile banking	Partial least squares	PEU and PU do not have significant effects on intention
[55]	Mobile government	User intention to adopt M-government	Structural equation modeling	PEU and PU have insignificant effects on adoption
[47]	Mobile banking	User intention to adopt mobile banking	Binary logistic regression	PEU and PU influence the successful adoption of mobile banking
[56]	Mobile-based services	Behavioral intention to use	Partial least squares	PEU and PU have significant effects on intention
[57]	Electronic banking	Reducing the problems/deficiencies in the use of electronic banking services	Regression analysis	With enhanced PEU, problems of using of electronic banking are decreased.

**Table 4:** Exploratory Factor Analysis Results.

Items	Factors			Cronbach’s Alpha
	1	2	3	
PT6	**0.613**	0.462	−0.080	0.662
PT1	**0.671**	0.363	−0.008	
PT7	**0.765**	0.340	−0.041	
PT3	**0.774**	0.154	0.042	
PS6	0.297	**0.598**	0.002	0.719
PS8	0.354	**0.707**	0.061	
PS1	0.174	**0.836**	0.073	
PC7	0.178	−0.087	**0.739**	0.663
PC8	0.022	0.036	**0.806**	
PC4	−0.064	0.192	**0.817**	

Bold indicates the factor groups with highest load values.

**Table 5:** CFA Goodness-of-fit Indexes.

Parameter	Value
Goodness-of-fit index (GFI)	0.906
Root-mean-square error (RMSEA)	0.077
Normed fit index (NFI)	0.891
Comparative fit index (CFI)	0.955
Relative chi-square (CMIN/DF)	1.591

**Table 6:** Developed Model.

Factors	Items
Perceived Security	To what extent are your operations protected from threats while using online banking (offence; attack; theft of money, documents, information, passwords, etc.)? My personal information is kept confidential while using offline traditional banking. Transactions conducted through ATMs are secure.
Perceived Convenience	A lot of time is needed to obtain online banking services. Using online banking requires a lot of knowledge. Using an ATM requires a lot of knowledge.
Perceived Trust	To what extent is online banking reliable as a system of banking service provision? To what extent is offline (traditional) banking reliable as a system of banking service provision? Offline traditional banking fulfills the commitments that it assumes. Online banking service is clear.

## Data Availability

The data used for this study are available upon request.

## Appendix A. Developed Questionnaire

**Factors**	**Guiding** **References**	**Items**	**Questions**
Perceived Trust (PT)		PT1	1.1 To what extent is online banking reliable as a system of banking service provision?
	[58,88]	PT2	1.2 To what extent is an ATM reliable as a system of banking service provision?
		PT3	1.3 To what extent is offline (traditional) banking reliable as a system of banking service provision?
		PT4	1.4 Online banking fulfills the commitments that it assumes.
		PT5	1.5 ATM fulfills the commitments that it assumes.
		PT6	1.6 Offline traditional banking fulfills the commitments that it assumes.
		PT7	1.7 Online banking service is clear.
		PT8	1.8 ATM service is clear.
		PT9	1.9 Offline traditional banking service is clear.
Perceived Security (PS)	[49,59]	PS1	2.1 To what extent are your operations protected from any threats while using online banking (offence; attack; theft of money, documents, information, passwords, etc.)?
		PS2	2.2 To what extent are your operations protected from any threats while using ATMs (offence, attack, theft of money, documents, information, passwords, etc.)?
		PS3	2.3 To what extent are your operations protected from any threats while using offline traditional banking (offence; attack; theft of money, documents, information, passwords, etc.)?
		PS4	2.4 My personal information is kept confidential while using online banking.
		PS5	2.5 My personal information is kept confidential while using ATMs.
		PS6	2.6 My personal information is kept confidential while using offline traditional banking.
		PS7	2.7 Transactions conducted through online banking are secure.
		PS8	2.8 Transactions conducted through ATMs are secure.
		PS9	2.9 Transactions conducted through offline traditional banking are secure.
Perceived Convenience (PC)		PC1	3.1 To what extent is using online banking convenient?
		PC2	3.2 To what extent is using an ATM convenient?
		PC3	3.3 To what extent is using offline traditional banking convenient?
		PC4	3.4 A lot of time is needed to obtain online banking services.
	[61,89]	PC5	3.5 A lot of time is needed to obtain ATM services.
		PC6	3.6 A lot of time is needed to obtain offline traditional banking services.
		PC7	3.7 Using online banking requires a lot of knowledge.
		PC8	3.8 Using an ATM requires a lot of knowledge.
		PC9	3.9 Using offline traditional banking requires a lot of knowledge.
Perceived Ease of Use (PEU)		PEU1	4.1 To what extent is the process of using online banking easy?
		PEU2	4.2 To what extent is the process of using an ATM easy?
		PEU3	4.3 To what extent is the process of using offline traditional banking easy?
		PEU4	4.4 Much time is needed to perform online banking operations.
		PEU5	4.5 Much time is needed to perform ATM operations.
		PEU6	4.6 Much time is needed to perform offline traditional banking operations.
	[62,83]	PEU7	4.7 Online banking is flexible to interact with.
		PEU8	4.8 ATMs are flexible to interact with.
		PEU9	4.9 Offline traditional banking is flexible to interact with.
Perceived Usefulness (PU)		PU1	5.1 To what extent does using online banking satisfy your needs of a banking service?
		PU2	5.2 To what extent does using ATM satisfy your needs of a banking service?
		PU3	5.3 To what extent does using offline traditional banking satisfy your needs of a banking service?
		PU4	5.4 Using online banking would help me to better manage and keep track of my finances.
		PU5	5.5 Using the ATM would help me to better manage and keep track of my finances.
	[83,90]	PU6	5.6 Using offline traditional banking would help me to better manage and keep track of my finances.
		PU7	5.7 Online banking increases my financial transaction effectiveness.
		PU8	5.8 ATMs increase my financial transaction effectiveness.
		PU9	5.9 Offline traditional banking increases my financial transaction effectiveness.
